# Elucidating CO_2_ Hydrogenation over In_2_O_3_ Nanoparticles using Operando UV/Vis and Impedance Spectroscopies

**DOI:** 10.1002/anie.202209388

**Published:** 2022-08-18

**Authors:** Marc Ziemba, Mariusz Radtke, Leon Schumacher, Christian Hess

**Affiliations:** ^1^ Eduard Zintl Institute of Inorganic and Physical Chemistry Technical University of Darmstadt Alarich-Weiss-Str. 8 64287 Darmstadt Germany

**Keywords:** CO_2_ Activation, In_2_O_3_, Operando Spectroscopy, Reaction Mechanism, Reverse Water-Gas Shift

## Abstract

In_2_O_3_ has emerged as a promising catalyst for CO_2_ activation, but a fundamental understanding of its mode of operation in CO_2_ hydrogenation is still missing, as the application of operando vibrational spectroscopy is challenging due to absorption effects. In this mechanistic study, we systematically address the redox processes related to the reverse water‐gas shift reaction (rWGSR) over In_2_O_3_ nanoparticles, both at the surface and in the bulk. Based on temperature‐dependent operando UV/Vis spectra and a novel operando impedance approach for thermal powder catalysts, we propose oxidation by CO_2_ as the rate‐determining step for the rWGSR. The results are consistent with redox processes, whereby hydrogen‐containing surface species are shown to exhibit a promoting effect. Our findings demonstrate that oxygen/hydrogen dynamics, in addition to surface processes, are important for the activity, which is expected to be of relevance not only for In_2_O_3_ but also for other reducible oxide catalysts.

## Graphical abstract

CO_2_ activation over In_2_O_3_ nanoparticle catalysts is consistent with redox processes, including oxidation by CO_2_ as the rate‐determining step, whereby hydrogen‐containing surface species are shown to exhibit a promoting effect. Besides surface processes, subsurface/bulk dynamics are shown to be essential for the activity in reverse water gas‐shift reaction, as revealed by operando UV/Vis and impedance spectroscopies.

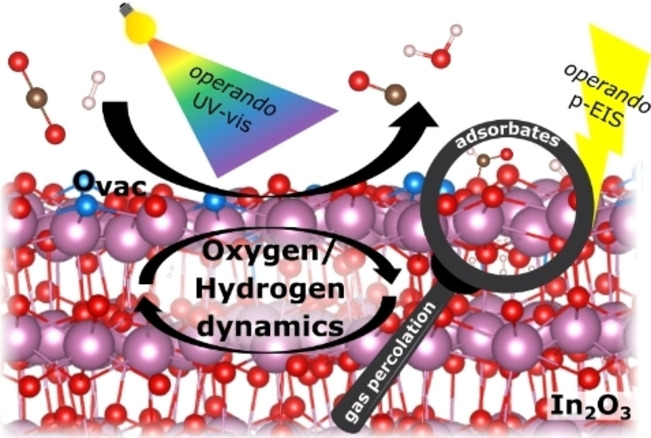

## Introduction

Catalysts based on cubic In_2_O_3_ (c‐In_2_O_3_, *Ia* $\bar{3}$ ) are known for their excellent properties in the context of CO_2_ activation, such as for methanol synthesis,[^1^, ^2^, ^3^, ^4^, ^5^, ^6^] direct liquid fuel production using bifunctional catalysts,^[7]^ or the reverse water‐gas shift reaction (rWGSR).[^8^, ^9^, ^10^, ^11^, ^12^] The latter reaction is of great relevance for the energy sector and the chemical industry, since CO_2_ can be converted to CO, which can then be hydrogenated to liquid fuels (via the Fischer–Tropsch process) or used as feedstock for chemical processes. Such an approach would allow the increasing energy demand to be met while reducing the large amounts of CO_2_ emitted by burning fossil fuels.

Theoretical studies have addressed CO_2_ hydrogenation over In_2_O_3_, highlighting the importance of oxygen vacancies,^[8]^ where H_2_ dissociation is thermodynamically and kinetically favored.^[13]^ More specifically, a frustrated Lewis pair (FLP) on the In_2_O_3−*x*_(OH)_*y*_(111) surface has been proposed to be responsible for H_2_ dissociation as well as for CO_2_ reduction, with the latter being the rate‐limiting step.[^14^, ^15^] Another theoretical (density functional theory, DFT) study^[6]^ on possible reaction pathways reports that the protonation of a bent CO_2_ adsorbate (bt‐CO_2_*) to a carboxylate intermediate (COOH*) is the rate‐limiting step for CO formation, which is followed by COOH* decomposition into CO and hydroxide on the surface.^[6]^ In a combined in situ spectroscopic and DFT study, the temperature‐dependent reduction behavior of c‐In_2_O_3_ was investigated.^[16]^

On the experimental side, surface indium oxo species have been proposed to be responsible for the heterolytic dissociation of hydrogen on alumina‐supported c‐In_2_O_3_, which is associated with the formation of surface indium hydrides and hydroxyl groups.^[17]^ Other studies on In_2_O_3_‐CeO_2_ catalysts highlight the importance of oxygen vacancies in In_2_O_3_ for CO_2_ conversion.^[9]^ Cubic In_2_O_3_ has been shown to be more suitable for rWGSR than hexagonal In_2_O_3_, by enhancing the dissociative adsorption of H_2_, facilitating the formation of oxygen vacancies and increasing the ability to adsorb and activate CO_2_.^[8]^ Defect‐rich In_2_O_3_ has been employed as a photocatalyst for rWGSR,[^18^, ^19^, ^20^] exhibiting high activities at ≤200 °C, whereas at higher temperatures the difference between photochemical and thermal activities became smaller.^[18]^

Mechanistic data on In_2_O_3_ during the thermal rWGSR (≥200 °C) is still very limited, in particular on the surface chemistry and subsurface/bulk dynamics, which may be related to experimental challenges for IR and Raman spectroscopy resulting from the In_2_O_3_ absorption properties.[^16^, ^21^, ^22^] In fact, to the best of our knowledge, so far no operando studies on the rWGSR over In_2_O_3_ catalysts have been reported. Nevertheless, (sub)surface/bulk processes seem to play an important role in c‐In_2_O_3_ catalysts, as our previous study^[16]^ has shown an exchange of oxygen vacancies between bulk and (sub)surface at elevated temperatures. Besides, diffusion of hydrogen may be of relevance by affecting the electronic structure[^23^, ^24^, ^25^] or the formation of hydrogenated intermediates (e.g. COOH*),[^6^, ^26^] thereby influencing the catalytic activity. Impedance measurements were employed to gain mechanistic insight in the context of methanol steam reforming and (r)WGS,^[12]^ but further analysis using equivalent‐circuit fitting or operando monitoring was not performed. In summary, it appears that in the literature both a regenerative redox mechanism (without the occurrence of reaction intermediates like e.g., carbonates, COOH*) and the reaction via intermediates are postulated, whereby the simultaneous occurrence is also conceivable.[^3^, ^6^, ^8^, ^21^, ^26^]

In this mechanistic study, we employ (quasi) in situ and operando spectroscopies (Raman, UV/Vis, X‐ray photoelectron spectroscopy (XPS)) to explore the surface and subsurface dynamics of In_2_O_3_ during rWGSR. Bulk properties are directly probed by operando impedance spectroscopy supported by ex situ X‐ray diffraction (XRD) analysis. We demonstrate, on one hand, the readiness of the In_2_O_3_ surface for reduction by H_2_ and oxidation by CO_2_, and, on the other, the participation of the subsurface/bulk in the reaction, thus enhancing the understanding of the mechanism, in particular, the CO_2_ activation process, the role of oxygen vacancies, and the participation of hydrogen‐related adsorbates.

## Results and Discussion

Details of the characterization of the In_2_O_3_ sample used in this study have already been published.^[16]^ Briefly, the particles have a specific surface area of 39 m^2^ g^−1^ and our ex situ XRD results show that only cubic (*Ia* $\bar{3}$ ) In_2_O_3_ is present. In addition, transmission electron microscopy (TEM) measurements show that the particles are present as sheets terminating with an In_2_O_3_(222) surface. Contaminations caused by the synthesis, e.g. nitrogen, can be excluded within the sensitivity of our XPS measurements.

**Figure 1:**
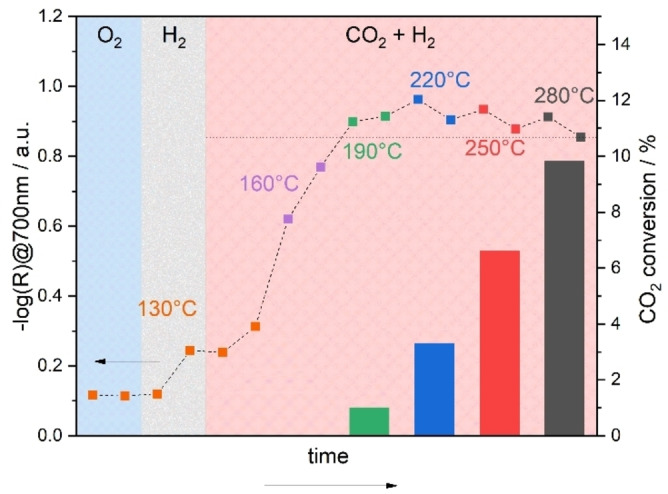
In situ/operando UV/Vis results for In_2_O_3_ sheets recorded during O_2_ and H_2_ exposures at 130 °C and reaction conditions (CO_2_:H_2_, 2 : 4) ranging from 130 to 280 °C in 30 °C steps. From 190 °C on, the sample becomes active in terms of conversion of CO_2_, which is shown by bars (right axis). At lower temperatures, the conversion is within the experimental uncertainty. The exposure time before the conditions were changed was ≈30 min. The horizontal dotted line helps to illustrate the change in absorbance in the reaction phase.

First, the In_2_O_3_ was analyzed for its activity as a function of temperature, while we simultaneously monitored the electronic structure using operando UV/Vis spectroscopy. Our previous UV/Vis studies on In_2_O_3_ during 10 vol % H_2_ exposure have revealed a strong dependence of the absorption in the visible range on the oxygen defect density.^[16]^ The bottom panel of Figure 1 depicts the temporal evolution of the absorbance at 700 nm as an indicator for oxygen defects upon exposure to different gas phases or temperatures. Starting at 130 °C, switching from O_2_ to H_2_ leads to an increase in absorption in the visible range and H_2_O formation in the gas phase, which illustrates that In_2_O_3_ undergoes reduction even at just 130 °C. Exposure to reaction conditions shows a further increase in absorbance, resulting from the longer residence time under H_2_ flow, since CO_2_ is not yet activated at this temperature, as evidenced by the absence of CO in the gas phase (not shown).

Increasing the temperature to 160 °C leads to a strong increase in absorption, which continues even after 160 °C is reached. This behavior can be explained by a strong reduction of the catalyst, as supported by the absence of CO but increasing concentration of H_2_O in the gas phase. In this context, H_2_‐temperature programmed desorption (TPD) experiments from previous studies^[21]^ have shown that a large fraction of adsorbed hydrogen is already desorbed at these temperatures thus does not remain on the surface.

Starting at a temperature of 190 °C, the catalyst becomes active towards CO_2_ conversion and shows only a weak increase or even a decrease in visible absorption. The absorption reaches its maximum during the first measurement at 220 °C and then decreases again over the next 30 min at 220 °C. The changes in absorption behavior can be explained in terms of counteracting contributions to the indium reduction state from temperature‐dependent reduction and CO_2_ conversion leading to oxidation. At 220 °C the maximum degree of reduction is reached under reaction conditions but at this temperature CO_2_ is significantly converted, which oxidizes the surface and thus results in an overall decrease in visible absorption. With time, a stationary state is established by balancing the contributions from reduction by H_2_ and oxidation by CO_2_. At temperatures >190 °C, a similar absorption behavior is observed, consisting of an initial increase and a subsequent decrease in absorption, which clearly shows that reduction by H_2_ is faster than oxidation by CO_2_. Comparison of the different equilibrated states reveals that the absorption is at its highest at 190 °C and decreases with temperature, strongly suggesting that the oxidation of the catalyst by CO_2_ increases with temperature while the reduction remains at about the same level, because the maximum amount of oxygen vacancies has been reached. This could be a first indication that CO_2_ activation with subsequent formation of CO is the rate‐determining step. The same experiment was performed with a CO_2_ to H_2_ ratio of 4 : 2 (see Figure S1). The results are similar, but the reduction is much slower due to the lower H_2_ content, which is why the further reduction of the material ends only after 250 °C. These findings underline our previous results.

The activities we measured compare favorably to those of previous studies on polycrystalline In_2_O_3_,[^9^, ^11^] which is likely to terminate with the 111 surface due to the method of synthesis (thermal decomposition of In(NO_3_)_3_) and the fact that it is the most thermally stable. However, this should be viewed with caution, as previously lower flow rates were used, resulting in higher residence times, and higher reactant concentrations in the gas feed. For this reason, we will refrain from a more detailed comparison of the activities. Other factors leading to higher conversions may be the smaller crystallite size and thus the larger specific surface area of the In_2_O_3_ particles used here. Besides, the particle shape or the surface termination may have an influence on the activity, as has been shown in the context of CO oxidation,^[27]^ while differences in H_2_ or CO_2_ activation over In_2_O_3_(111) or In_2_O_3_(110) have been demonstrated by theoretical studies (DFT).[^6^, ^13^, ^28^, ^29^]

**Figure 2:**
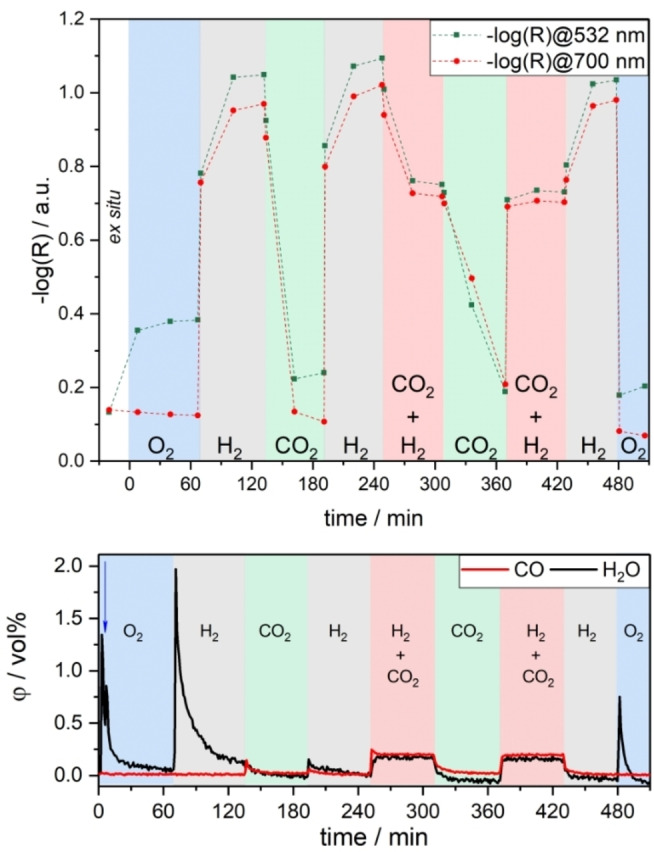
Top: In situ/operando UV/Vis results for In_2_O_3_ sheets recorded during the indicated gas exposures at 250 °C and at a total flow rate of 100 mL min^−1^, except for the ex situ spectra, which were taken at 25 °C. The exposure time in each gas phase was about 1 h with the exception of the last O_2_ phase (30 min). Bottom: Gas‐phase IR analysis during the UV/Vis measurements. The increased water concentration in the first few minutes is due to the purging of the cell. Starting after 6 min (see blue arrow) the cell was heated to 250 °C, leading to water desorption and thus an increase in the H_2_O concentration. For details see text.

Next, we performed operando UV/Vis measurements at 250 °C while systematically varying the gas phase between oxidative/reactive and reductive conditions. Figure 2 shows the absorbances at 532 nm and 700 nm (see Figure S2 for the corresponding UV/Vis spectra), where the former mainly resembles the self‐absorption in our Raman experiments, which were recorded at 532 nm (see below). In these experiments we first pretreated the catalyst with O_2_ to start from an oxidized state. During heating to 250 °C under O_2_ exposure, desorption of H_2_O and CO_2_ was observed (not shown), indicating that the surface is being cleaned from adsorbates. Upon exposure to O_2_ flow, the absorption at 532 nm increased significantly (see Figure 2). Since this wavelength is located within the absorption edge, the observed behavior can be explained by an increase in the band edge, originating from the temperature increase (Burstein–Moss effect) and/or from oxidation.^[16]^

On switching to H_2_, the absorption at 532 and 700 nm increases significantly, which is mainly attributed to a reduction of the (sub)surface and possibly also to an increase in reflectivity through small metallic indium domains. This is supported by the strong presence of H_2_O in the gas phase. Since the same trends in absorption can be observed at both wavelengths (and the entire remaining visible range), only the absorption at 700 nm will be discussed in the following.

Upon exposure to CO_2_, there is again a strong decrease in absorption, which is in the range of the absorption in O_2_, demonstrating that the sample can be oxidized again by CO_2_ and that the process of reduction seems to be completely reversible on the basis of UV/Vis spectra and their penetration depth. At the same time, the gas phase initially shows a small increase in CO (see Figure 2, bottom panel), implying surface oxidation by CO_2_, thereby releasing CO. Such a reversibility with respect to the oxidation state in the absence of H_2_ could indicate that hydrogen is not required for CO_2_ reduction.

On switching to H_2_ afterwards, the detected CO signal is significantly lower, which shows that only small amounts of CO_2_ in the form of carbonates or other carbonaceous adsorbates remain on the surface in the CO_2_ phase. In contrast, the H_2_O signal increases, which in turn is associated with the reduction of the surface, as can be seen by the increase in absorbance.

Exposure to reaction conditions (H_2_/CO_2_) induces a decrease in absorption despite the same H_2_ concentration, which, however, does not reach the level detected in CO_2_. This behavior confirms the findings of Figure 1, i.e., that the reduction and oxidation processes must be in equilibrium, with the reduction still predominating. Interestingly, the two reaction phases are characterized by the same absorption (see Figure 2, top panel) and CO evolution (see Figure 2, bottom panel), despite the completely different initial states. This clearly demonstrates that the pretreatment has no influence on the reaction and suggests that certain intermediates only form under reaction conditions, since the CO release under reaction conditions is significantly higher than when switching from H_2_ to CO_2_ or vice versa. In this context, previous photocatalytic studies have shown that the surface hydroxide concentration is also higher under reaction conditions than under pure H_2_.^[18]^ As can be seen in Figure 2, switching off CO_2_ after reaction conditions leads to a renewed increase in absorption and thus a reduction of the surface.

Finally, In_2_O_3_ is exposed to O_2_ flow to check the reversibility of the system. The gas‐phase data reveals a strong H_2_O signal decaying with time, which implies the presence/formation of hydroxides/adsorbed hydrogen during H_2_ exposure. During this O_2_ exposure, the absorbance is lower than during the first O_2_ phase, and major differences were detected for 532 nm absorption, which is located in the absorption edge. Since in both cases the experimental conditions were the same, we attribute the observed drop in absorption to a decrease in the band gap resulting from a larger crystallite size. To test this hypothesis, we performed ex situ XRD measurements after different gas exposures until the first reaction phase, which clearly show that the crystallite size increases from 12 nm after the first O_2_ phase to 27 nm after the subsequent H_2_ (for diffractograms, see Figure S3). Note that all following gas atmospheres have no significant influence on the crystallite size. Notably, after the second H_2_ exposure, additional reflections are detected, which originate from metallic indium (JCPDS 85‐1409), showing that metallic indium is formed during reduction with 4 vol % H_2_ at 250 °C. To this end, it should be noted that small domains of metallic indium, formed during the first H_2_ phase and under reaction conditions, may have oxidized back under air exposure and thus cannot be excluded by the present ex situ XRD analysis.

**Figure 3:**
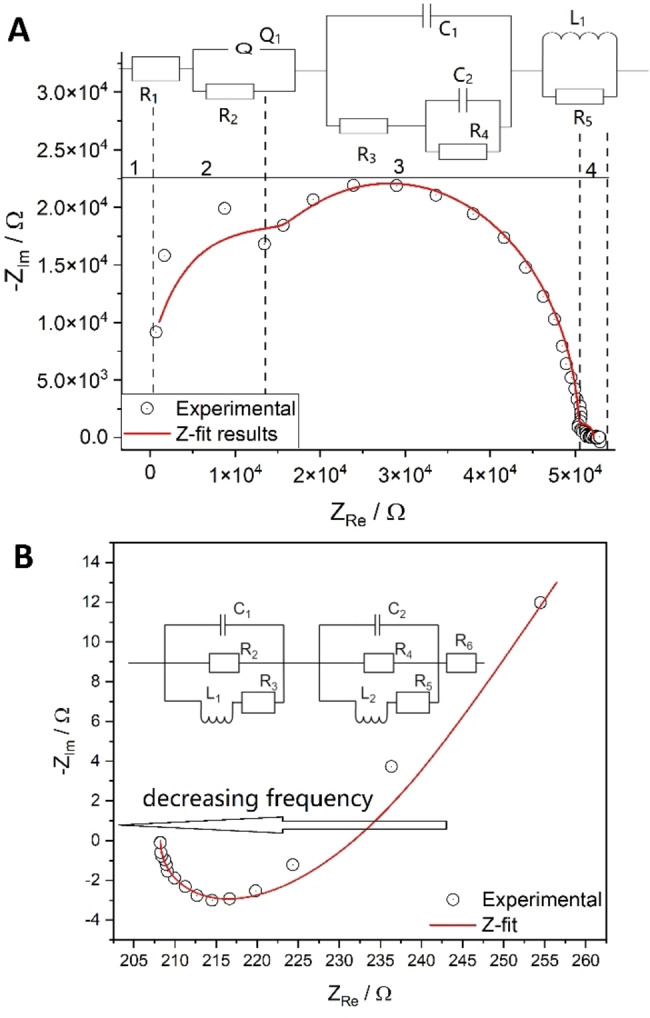
Nyquist plots based on operando p‐EIS of In_2_O_3_, shown together with fits according to the corresponding equivalent circuits. A) During CO_2_ gas treatment (2 vol %; total flow: 50 mL min^−1^) at 250 °C with the different areas in the Nyquist plot and the corresponding assignment in the equivalent circuit. Region 1: electrode bulk resistance; region 2: vacancy reorganization; region 3: capacitive behavior of the non‐reduced In_2_O_3_; region 4: gas diffusion into In_2_O_3_ nanoparticles. B) During CO_2_/H_2_ treatment (2 vol % CO_2_, 4 vol % H_2_; total flow rate: 50 mL min^−1^) at 250 °C. The experimental data shows a reversed behavior, with high‐frequency points located on the right. R: resistance, C: capacitance, L: inductance, Q: non‐ideal capacitance. The values resulting from the fit analysis are summarized in Table S2.

To further probe the reducibility behavior of In_2_O_3_, we recorded the XRD pattern after CO exposure (4 vol %) after a prior oxygen treatment, analogous to the other measurements. For CO exposure, significantly more metallic indium was formed and the crystallite size grew only to 21 nm, in contrast to the observed 27 nm for H_2_. Thus, hydrogen is a weaker reducing agent towards In_2_O_3_ than CO, which is consistent with the literature.^[12]^ Furthermore, hydrogen must have an additional effect on the sample, since the crystallite size increases significantly more compared to CO. To gain insight into the electronic structure changes during CO reduction, we recorded in situ UV/Vis spectra (see Figure S4), which reveal a higher absorption in the visible region during CO exposure, implying a better reduction by CO. We also highlight the fact that the presence of CO strongly contributes to the enhanced electronic inductive effects in the impedance spectra in the Figure 3B compared to the purely reductive H_2_ treatment (Figure S6, Table S2). Interestingly, the sample absorbance almost returns to its original value (measured in O_2_) on subsequent exposure to CO_2_. Thus, In_2_O_3_ can be re‐oxidized by CO_2_, releasing CO regardless of the reducing agent (H_2_ or CO). Moreover, no surface hydrogen species are required for the oxidation by CO_2_, indicating that no hydrogen‐containing intermediates are involved in the re‐oxidation process.

To elucidate the charge and mass transport in In_2_O_3_ nanoparticles, operando potentiostatic electrochemical impedance spectroscopy (p‐EIS) spectra were acquired. It should be mentioned that due to the sample shape (no pellet) a Mott–Schottky analysis is disturbed by gas inclusion and an increased number of grain boundaries and is not completely reproducible. For this reason, such an analysis was not performed in this work. The non‐uniform sample distribution also has an impact on the p‐EIS spectra, which deviate from ideal behavior by ca. 10 %, as validated by Kramers‐Kronig‐relations (see Supporting Information). All spectra (see Figure 3 and Figures S5–S11) were recorded under the same gas sequence and composition as in Figure 2. A considerable impact of the gas phase on the impedance spectra was found for all treatments, which is reflected in the profile of the Nyquist plots. However, as there are no fundamental differences between spectra taken under the same gas atmosphere, only the spectra of the first treatment with a specific gas phase will be discussed, starting with the spectrum under O_2_ exposure (see Figure S5).

As In_2_O_3_ is a semiconductor with a band gap of approximately 3 eV,^[30]^ accumulation of charged surface oxygen due to polarization is expected.^[31]^ The polarization manifests itself in a sharp and vertical increase of the impedance in the Nyquist plot, and both imaginary and real parts of the impedance reach values in the megohm range (see Figure S5), which speaks for very limited to no conductivity of In_2_O_3_ during the oxygen treatment. The spectrum in O_2_ atmosphere may therefore be treated as a response of bare In_2_O_3_ nanoparticles and will serve as a reference for the discussion of changes observed during the following gas treatments.

Regarding surface charging, a similar behavior was previously observed for CeO_2_ at room temperature.^[32]^ The presence of a limited diffusion region results from the hindered oxygen accumulation on the In_2_O_3_ surface. The voltage applied to the sample causes external polarization due to the non‐conductive character of In_2_O_3_ at room temperature, and the activation energy from the temperature dependent conductivity behavior of 137 kJ mol^−1^ will not be reached by simple polarization but requires elevated temperatures.^[33]^

The major reduction of In_2_O_3_ was observed during the hydrogen treatment (see Figure S6). The pronounced contribution of the inductive part (*L*=2.03×10^−5^ H during the first H_2_ treatment) observed in the operando p‐EIS spectrum is attributed to the percolation of In_2_O_3_ nanoparticles with hydrogen species,^[34]^ referring here to the penetration of these species into the solid. This is in agreement with previous IR and DFT studies on In_2_O_3_, which have demonstrated the diffusion of interstitial hydrogen to increase conductivity.[^23^, ^24^, ^25^] Thereby, hydrogen percolates into the bulk of a nanoparticle and creates an inductive response of the EIS spectrum, typical for gas diffusion in the bulk of a material.^[34]^ The reduction of the In_2_O_3_ nanoparticles is proposed to proceed according to the reaction In_2_O_3_+3 H_2_→2 In^0^+3 H_2_O. Thus, the first two H_2_ phases show a purely resistive behavior at the beginning of the Nyquist plot, indicating the formation of metallic indium. In the third phase, this behavior is less pronounced, but this is also consistent with our UV/Vis results (lowest absorption at 532 nm).

The decomposition of H_2_ into protons within In_2_O_3_ was speculated to occur by a homolytic or a heterolytic splitting process, as discussed by García‐Melchor and López.^[35]^ In this context, theoretical studies have shown that both scenarios are conceivable, but strongly depend on the degree of reduction.^[13]^ Under our conditions, owing to the high degree of indium oxide reduction, heterolytic dissociation is more likely.^[13]^ To describe the spectrum in Figure S6, a considerable contribution of the Warburg element is necessary (see Table S2), which speaks for the distribution of hydrogen gas on the surface. The Warburg element is commonly used to describe mass transport processes, therefore, coupling it with inductive parts will deliberately lead to the description of a gaseous contribution to the impedance response.^[36]^

Following the hydrogen treatment, In_2_O_3_ was exposed to CO_2_ and an operando p‐EIS spectrum was recorded (see Figure 3A). The equivalent circuit shown in Figure 3A is divided into four parts. The first part represents the electrode bulk resistance (*R*_1_), which was found to be in the ohm range (see Table S2), speaking for improved conductivity compared to the pristine material (with *R*_1_ in the megohm range); nevertheless, remaining vacancies induced by the H_2_ phase remain active, judging by our Raman spectra below. In the second part, the reorganization of vacancies occurs.^[37]^ The third part contributes mostly to the p‐EIS spectrum and is assigned to non‐reduced In_2_O_3_, while at low frequencies (L_1_R_5_ region) gas diffusion takes place, as, for example, observed for polycrystalline tin oxide.[^38^, ^39^] The regions are assigned based on the frequency domains where impedance data was recorded, starting from the high‐frequency region and bulk impedance, followed by the internal electronic structure in the middle‐frequency region and ending on the diffusion response within low‐frequency regions.^[40]^

During reaction conditions (see Figure 3B), the hydrogen flow, penetrating the interior of the In_2_O_3_, carries the CO_2_ gas with it, as judged by the inductance contribution (*L*=1.03×10^−5^ H), which differs from the value obtained for the first H_2_ treatment (*L*=2.03×10^−5^ H). The reaction occurs in the bulk due to gas percolation, rather than on the surface as in the case of pure CO_2_ exposure. In this context, we propose that the higher inductance is caused by the reaction CO_2_+H_2_→CO+H_2_O and thus by the presence of CO, which is also detected in the gas phase. Therefore, the respective parts of the triple equivalent circuits of Figure 3B correspond to the interaction of In_2_O_3_ with H_2_ (right part of the circuit) and CO_2_ (left part of the circuit). Please note that the EIS spectrum is reversed compared to what it was in the case of H_2_ treatment, implying that the high frequency points are in the region of high imaginary and real impedances, originating from gas percolation and the induction of the gas impedance.^[34]^ In contrast to pure hydrogen, there is no pure resistance behavior at the beginning of the spectrum, indicating that no metallic indium is formed under reaction conditions. This is corroborated by our operando UV/Vis results, which show a significantly lower absorption in the visible region than under H_2_ atmosphere.

**Figure 4:**
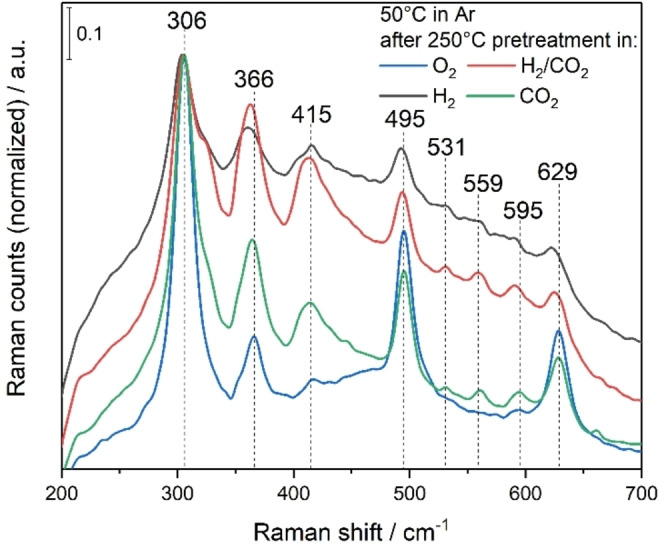
Quasi in situ Raman spectra recorded in argon at 50 °C after the indicated gas exposures at 250 °C and at a total flow rate of 100 mL min^−1^. The spectra were normalized to the band with the highest intensity at around 306 cm^−1^. The spectra were recorded sequentially in the order O_2_, H_2_, H_2_/CO_2_, and CO_2_. The sample was cooled to 50 °C under argon.

Figure 4 depicts quasi in situ Raman spectra of In_2_O_3_, recorded under argon at 50 °C after treatment with O_2_, H_2_, H_2_/CO_2_, and CO_2_. The bands at 306, 366, 495 and 629 cm^−1^ can be assigned to δ(InO_6_), ν(InO_6_), In−O−In, and ν(InO_6_) vibrations of cubic In_2_O_3_, respectively, which is in accordance with the literature.[^16^, ^41^] These bands become broader with increasing number of defects (see H_2_ and H_2_/CO_2_ treatment, Figure 4), consistent with previous studies.^[42]^ The feature at 595 cm^−1^ also originates from cubic In_2_O_3_ and is attributed to a vibration with *E*_g_ symmetry.^[43]^ It shows a red‐shift under reaction conditions and H_2_, as a result of defect formation, consistent with our previous study.^[16]^ All other features (415, 531, and 559 cm^−1^) are related to oxygen defects, as confirmed by DFT.^[16]^ Notably, the band at 306 cm^−1^ shows a shoulder towards larger wavenumbers, which is caused by reduction and was also observed in previous studies.[^21^, ^42^, ^44^]

Comparison of the different gas phases reveals a significantly higher background for H_2_ and reaction conditions, which is caused by fluorescence, self‐absorption of the laser, and an increased reflectivity by metallic indium (as also detected by p‐EIS, see flat line for *Z*_Re_ between 240 and 220 Ω in Figure S6). Nevertheless, closer inspection of the spectra recorded after O_2_ and CO_2_ exposure shows that the 415 cm^−1^ band is less pronounced for O_2_, implying that re‐oxidation by CO_2_ is not as efficient as by oxygen. This behavior is in agreement with previous impedance studies,^[12]^ which show that In_2_O_3_ is best re‐oxidized with O_2_, followed by H_2_O and CO_2_. In the UV/Vis measurements such a difference was not observed due to the lower penetration depth, implying that the surface is completely oxidized, but the bulk is not. Thus, a certain number of defects remain present even under O_2_.

The high‐wavenumber region of the Raman spectra shows bands at 3647 and 3675 cm^−1^ (see Figure S12), which originate from bridging hydroxyl groups[^44^, ^45^] and reappear after exposure to reaction conditions after being consumed in the reducing atmosphere. Interestingly, after reaction conditions, a new band is detected at 2867 cm^−1^, which is assigned to the C−H stretching vibration of a formate‐like species.^[44]^ This shows that under reaction conditions a stable adsorbate is formed, which involves atoms from both reactants. However, in this context it is important to note that this observation does not imply the presence of a reaction intermediate, as the detected band may be due to an observer species. At this point it should be noted that we cannot exclude the presence of other intermediates. Future operando Raman experiments may possibly clarify the role of formate or other species, but this is currently hampered by the large background (see introduction).

**Figure 5:**
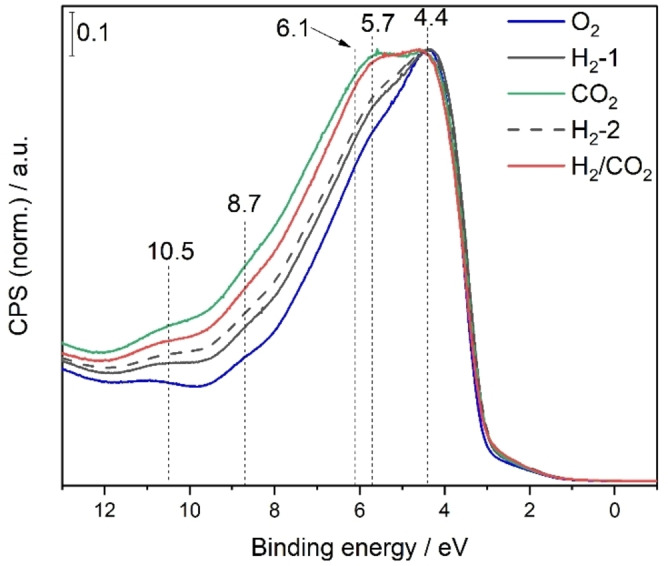
Valence band photoelectron spectra recorded quasi in situ after exposure to the indicated gas atmospheres at 250 °C and at a total flow rate of 100 mL min^−1^. The blue spectrum was obtained after O_2_ (25 %) pretreatment, the gray ones after H_2_ (4 %) pretreatment, the green one after CO_2_ (2 %) pretreatment, and the red one after H_2_/CO_2_ (4 %/2 %) pretreatment. The gas sequence corresponds to that of the UV/Vis experiments in Figure 2 up to the first reaction phase and the hyphenated number indicates the order of the gas sequence. The source was He I (*hν*=21.2 eV).

In order to obtain additional surface information, XPS and ultraviolet photoelectron spectroscopy (UPS) was applied (for XPS data and discussion see Supporting Information). Figure 5 depicts the UP spectra of In_2_O_3_ using He I radiation after exposure to the different gas atmospheres, analogous to the XP spectra. All UP spectra show the typical valence band features at 4.4, 6.1 and 8.7 eV.[^46^, ^47^] For all gas atmospheres, the features at 5.7, 8.7, and 10.5 eV increase in intensity compared to the spectrum after O_2_ exposure, but most prominently for exposure to CO_2_ and reaction conditions. Previous studies have proposed the contributions at 5.7 and 10.5 eV to be determined by modified valence band (VB) states subjected to adsorption of molecules.^[46]^ In our case, in principle, hydroxides and/or carbonaceous adsorbates (e.g., carbonates, formats) may contribute to the spectral changes compared to the O_2_ spectrum, but the presence of carbonaceous adsorbates appears to be more likely, considering the increased intensity after exposure to CO_2_ and the reaction phase. This is further supported by the IR gas‐phase measurements, which show the presence of hydrogen‐containing adsorbates on the surface after H_2_ treatment (see Figure 2). In this context, in a previous near‐ambient‐pressure (NAP) UPS study on In_2_O_3_ during rWGSR, it was shown that the temperature as well as the CO_2_:H_2_ ratio have a strong influence on the valence region,^[48]^ which is reflected in line broadening. This is in agreement with our findings that the surface is strongly influenced by the gas environment, where a greater CO_2_ content leads to greater changes, which indicates the presence of carbonaceous adsorbates.

Summarizing the findings from the combined surface analysis by Raman, XP and UP spectroscopies, there is a clear indication of variations in the surface composition, the indium state, and the number of oxygen vacancies when switching between oxidative, reductive, and reactive gas atmospheres. The indium and support surface state after exposure to reaction conditions largely follows a behavior expected within the limits of oxidative (CO_2_) and reductive (H_2_) atmospheres, while the overall changes in the adsorbate species (e.g. OH, formate) are rather distinct, somewhat resembling the observed subsurface/bulk behavior (see Figure 2). By combining these findings with the operando UV/Vis and impedance results, the active state of the catalyst can be specified as reduced, i.e., oxygen‐defect containing (but non‐metallic) In_2_O_3_, percolated with reactant and product gases, and exhibiting surface species, such as hydroxides, hydrides, and carbonaceous adsorbates. Although the redox processes can occur separately, the simultaneous presence of H_2_ and CO_2_ clearly provides synergetic effects towards CO_2_ conversion. Our results emphasize, in particular, the importance of subsurface/bulk dynamics for the activity in reducible oxide catalysts, besides surface processes.

## Conclusion

In this study, we have generated a deeper understanding of the mechanism of rWGSR over c‐In_2_O_3_ catalysts by applying operando UV/Vis spectroscopy and (in the context of thermal catalysis) newly developed operando impedance spectroscopy, as well as quasi in situ spectroscopies (Raman, XPS, UPS). The new mechanistic insight into the In_2_O_3_ mode of operation was supported by a systematic investigation of the related reduction and oxidation processes and their comparison to reaction conditions. For example, our study shows that despite surface reduction, diffusion of oxygen vacancies leads to bulk reduction. While reduced c‐In_2_O_3_ nanoparticles can be re‐oxidized by CO_2_ on the surface, complete oxidation of the bulk is only possible with O_2_. This behavior is independent of the reducing agent (H_2_ or CO), although CO leads to stronger In_2_O_3_ reduction.

Based on temperature‐dependent UV/Vis analysis under reaction conditions, oxidation by CO_2_ is identified as the rate‐determining step for the rWGSR, which is supported by operando impedance spectra, showing a predominance of the hydrogen contribution. The significant evolution of CO and H_2_O, also after separate treatment with CO_2_ or H_2_, is fully consistent with redox processes but because conversions are somewhat higher under reaction conditions, hydrogen‐containing surface species (e.g. hydroxides, hydrides) are proposed to have a promoting effect on CO_2_ conversion and/or hydrogen containing intermediates (e.g. COOH*) may play an role. Such a behavior would be in accordance with results from theoretical studies, which associate CO_2_ conversion with frustrated Lewis pairs.[^14^, ^15^]

The above analysis of the (sub)surface and bulk properties during the rWGSR demonstrates that the entire In_2_O_3_ nanoparticle is involved in the reaction, which is only accessible by using complementary in situ/operando techniques. Combining our new experimental findings with results from theory allows us to develop a consistent mechanistic picture of rWGSR over c‐In_2_O_3_. Our findings are of immediate relevance for a detailed understanding of CO_2_ hydrogenation and other related catalytic processes over In_2_O_3_, but they are also expected to be of more general impact, considering the growing importance of catalysts based on reducible oxide materials.

## Conflict of interest

The authors declare no conflict of interest.

1

## Supporting information

As a service to our authors and readers, this journal provides supporting information supplied by the authors. Such materials are peer reviewed and may be re‐organized for online delivery, but are not copy‐edited or typeset. Technical support issues arising from supporting information (other than missing files) should be addressed to the authors.

Supporting Information

## Data Availability

The data that support the findings of this study are available from the corresponding author upon reasonable request.
