# Supplementary material for: Elucidating CO2 Hydrogenation over In2O3 Nanoparticles using Operando UV/Vis and Impedance Spectroscopies
Source: Angew Chem Int Ed Engl. 2022 Aug 18;61(39):e202209388. doi: 10.1002/anie.202209388 (PMC9805051; doi:10.1002/anie.202209388)
Supplement: Supplementary file 1 — Supporting Information [file ANIE-61-0-s001.pdf]

Angewandte Chemie
Eine Zeitschrift der Gesellschaft Deutscher Chemiker

Supporting Information

Elucidating CO_2 Hydrogenation over In_2O_3 Nanoparticles using Operando UV/Vis and Impedance Spectroscopies

*M. Ziemba, M. Radtke, L. Schumacher, C. Hess**

Supplementary Experimental Section

Catalyst Preparation

c-In_2O_3 sheets were synthesized by calcination of $In(OH)_3$ at 600 °C, as described previously.[1] Briefly, $In(NO_3)_3 \cdot xH_2O$ (Alfa Aesar, UK, ≥ 99.99%) was dissolved in deionized water (in-house, reverse osmosis), which was then set to a pH value of 10 by using an ammonia solution (Carl Roth, Germany, ≥ 25 %). The precipitated $In(OH)_3$ was filtered off and washed five times with deionized water. The residue was dried in an air stream and calcined in air at 600 °C for 2 h at a heating rate of 10 °C/min. Afterwards, the obtained c-In_2O_3 particles were sieved using a 200 µm aperture sieve.

Catalyst Characterization

The catalytic activity as well as the operando UV-vis, in situ Raman and quasi in situ X-ray photoelectron (XP) spectroscopic measurements were performed using an experimental setup that has been described previously.[2–6] For all measurements, 20–25 mg of sample was placed in a stainless-steel sample holder (diameter: 8 mm; depth: 0.5 mm) and exposed to a gas flow consisting of 25 vol% O_2, 4 vol% H_2, 2 vol% CO_2 or a mixture of H_2/CO_2 (4 vol%/2 vol%) balanced with argon (total flow rate: 100 mL/min) at the given temperature. The balanced argon stream served to limit the conversion and to eliminate the influence of any mass-transport effects, thus allowing us to focus on the reaction mechanism. Furthermore, due to the cell geometry and since the feed gases flowed over the catalyst sample, the amount of catalyst had hardly any influence on the overall activity when the sample holder was completely covered. Details of the specific measurements are briefly described in the following sections.

Operando UV-vis Spectroscopy. UV-vis spectra were acquired in diffuse reflection modus using an AvaSpec ULS2048 spectrometer (Avantes, Netherlands), equipped with a separate deuterium lamp and a halogen discharge lamp. The measuring time was set as 60 s, which is composed of an exposure time of 300 ms and 200 runs. As the white standard, barium sulfate powder ($BaSO_4$, Sigma Aldrich, Germany) was used, which exhibits no absorption between 170 nm and 1100 nm. At the same time as these measurements, the gas phase was measured with a Fourier transform infrared (FTIR) spectrometer, as described previously.[4,6,7] Briefly, the resolution was 4 cm^{-1}, and the measurement time was 60 s, which allowed the accumulation of 125 spectra. By using calibration curves, the concentration of CO (and H_2O) was calculated in order to determine the conversion of CO_2. For the calculations, representative bands of the

gases H_2O and CO were integrated. The position of the maxima of the used bands are given in Table S1. The catalytic activity (CO_2 conversion) is defined as the ratio of the amount of CO measured by the FTIR spectrometer at the outlet of the cell to the amount of dosed CO_2. Based on our IR analysis, our selectivity for CO is 100%. To check the stability of the catalyst, we exposed the catalyst to reaction conditions (250 °C, 4 vol% H_2, 2 vol% CO_2, Ar) for over 5 hours but did not see any indication for instability or change in activity.

Table S1: IR band maxima of gas phase H_2O and CO used for the determination of the catalytic activity in the framework of the operando measurements.

Gas	IR band maxima / cm^{-1}
H_2O	1508
CO	2174

Quasi in situ Raman Spectroscopy. Raman spectra were recorded on an HL5R transmission spectrometer (Kaiser Optical Systems Inc., USA), which employs a frequency-doubled Nd:YAG laser (Cobolt Inc., Hübner Photonics GmbH, Germany) for excitation at 532 nm, as described previously.[8,9] For the measurements, a ½ inch immersion probe with sapphire optics (Kaiser Optical Systems Inc., US) was applied to the reaction chamber. The stability of the band positions is better than 0.3 cm^{-1}. The laser power at the position of the sample was set to 6 mW, with a spot size approximately 30 μm in diameter. After gas treatment, the sample was cooled to 50 °C in argon (total flow: 100 mL min^{-1}) for the Raman measurements, which were set to an exposure time of 900 s and two accumulations. For each measurement a cosmic ray filter was used, with a second spectrum recorded at each accumulation to eliminate spikes caused by cosmic rays, resulting in a total measurement time of about 1 h. The corresponding dark spectrum was recorded at the beginning of the measurement series. In this context, it should be mentioned that a separate dark spectrum before each individual measurement does not lead to any significant differences.

X-Ray Photoelectron Spectroscopy (XPS). XP spectra were recorded on a modified LHS/SPECS EA200 magnetic circular dichroism (MCD) system described previously.[1,2,7] The XPS system was equipped with a Mg Kα source (1253.6 eV, 168 W) and the calibration of the binding energy scale was performed with Au $4f_{7/2}$ = 84.0 eV and Cu $2p_{3/2}$ = 932.67 eV signals from foil samples. Prior to the measurements, the sample was treated with different gas atmospheres at 250°C, and the subsequent transfer of the sample into the analysis chamber was

performed without any air exposure (quasi in situ). Sample charging was compensated by setting the peak of the C 1s signal to 284.8 eV. Survey spectra were recorded at a resolution of 0.4 eV and narrow scans at a resolution of 0.025 eV. The O:In ratios in Figure S13 were obtained by integrating the In $3d_{5/2}$ and the O 1s signals after a Shirley background subtraction from the detailed spectra. The resulting areas were corrected with the corresponding relative sensitivity factors, i.e., 3.9 for the In $3d_{5/2}$ signal and 0.66 for the O 1s one.[10]

Ultraviolet Photoelectron Spectroscopy (UPS). UP spectra were recorded on our modified LHS/SPECS EA200 MCD system (see XPS). As UV source we used a UVS 10/35 (SPECS) in combination with helium. The gas inlet was controlled by a differential pumping system consisting of a rotary pump and a turbomolecular pump. The first pumping stage involved the rotary pump (10^{-2} mbar) and the second pumping stage was a simultaneous combination of both the rotary and turbomolecular systems (5×10^{-7} mbar). The calibration of the binding energy scale was performed with a gold foil (Alfa Aesar, UK, 99.985%) after Ar^+ ion sputtering. The UP spectra were recorded at a narrow-scan resolution of 0.025 eV.

X-Ray Diffraction (XRD). Powder XRD patterns were aquired on a Stoe Stadi P diffractometer with a Ge(111)-monochromator, Cu Kα radiation (λ = 1.54060 Å), and a MYTHEN-1K Dectris detector, using a flat sample holder in transmission geometry. The powder XRD patterns were recorded (ex situ) after the indicated gas treatment (1 h) at 250 °C (total flow: 100 mL/min).

Operando Impedance Spectroscopy. Potentiostatic electrochemical impedance spectra (p-EIS) were acquired in a two-electrode system using a BioLogic VSP potentiostat/galvanostat operated in the 1 MHz to 10 Hz range with 50 mV amplitude and 10 measurements points per decade acquired in triplicate in a potential range of 0.05–1 V versus a reference of +0.338 V (In^{3+} to In^0).[11] The positive reduction potential was referenced against the standard hydrogen electrode (SHE) and the potential interval was set to dE = 0.095 V. Impedance spectra were recorded in a commercial CCR1000 cell (Linkam Scientific Instruments, UK), equipped with a PTFE plate with two holes for the copper electrodes (see Scheme S1). In this context, we also performed experiments with gold electrodes (Alfa Aesar, UK, 99.999 %), where no influence of electrode material on the electrochemical output was observed with the exception of a parasitic potential iR drop, arising from the peculiarities of cell assembly. Its compensation was performed manually in the EC-Lab v. 11.33 (BioLogic, France) acquisition software prior to

the actual measurement. Before each measurement, the sample was kept at 250 °C for about 40 min in 25 vol% O_2, 4 vol% H_2, 2 vol% CO_2, or a mixture of H_2/CO_2 (4 vol%/2 vol%) balanced in argon at a total flow rate of 50 mL/min for equilibration. This procedure ensures that the measurements take place in a stationary state. This is verified by considering the temporal evolution, which for the respective potential did not show any significant changes during the measurement. Raw spectra were validated by applying the Kramers–Kronig relations, which deviate from ideal behavior by ca. 10%, with 11% being the benchmark for discarding the measurement, meaning that the real and imaginary parts of the experimental spectra overlap with the e.g. imaginary spectral points calculated from e.g. real part by applying Hilbert transforms. The impedance models were built in order to fit the acquired p-EIS spectra by the numerical Fortran-assisted Z-fit procedure (EC-Lab v. 11.33). For the fit analysis a numerical randomization followed by the Levenberg–Marquardt algorithm was utilized, while the number of iterations was kept at 500k to ensure full conversion of the equivalent-circuit model (error threshold: $\chi/|Z| < 0.5$).

At the same time as the impedance measurements, the gas phase was measured using a FTIR spectrometer (Tensor 20, Bruker). The settings correspond to those of the operando UV-vis measurements. The CO_2 conversion during the reaction phases was estimated to be 4 %.

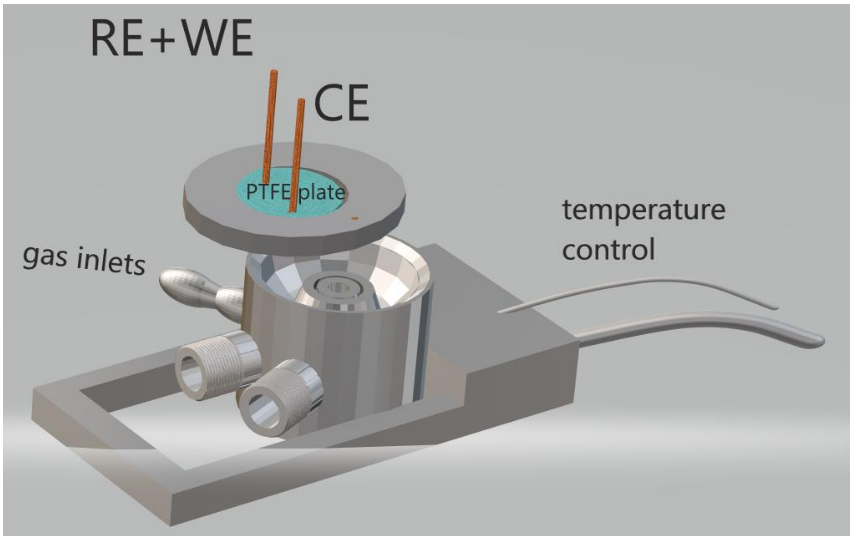

Scheme S1. Scheme of the experimental setup used for the impedance measurements. RE: reference electrode, WE: working electrode, CE: counter electrode. For more details see text.

Supplementary Figures and Table

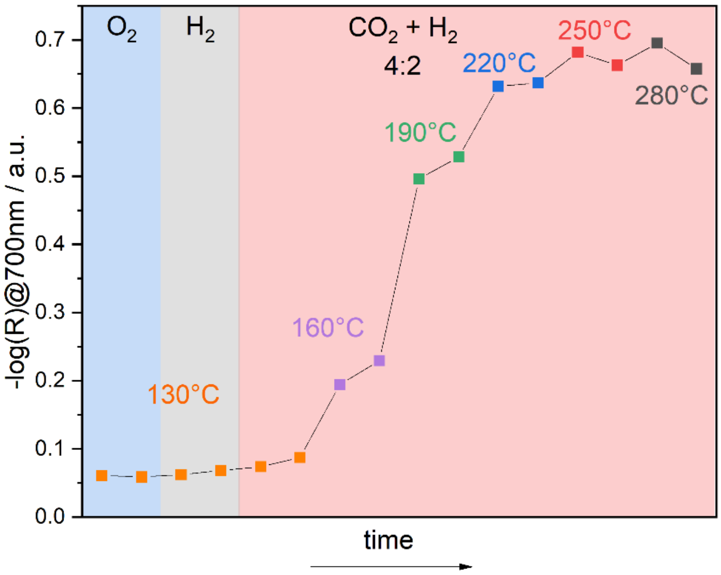

Figure S1. In situ/operando UV-vis results for In_2O_3 sheets recorded during O_2 (25 vol%) and H_2 (2 vol%) exposures at 130 °C and reaction conditions (4 vol% CO_2/2 vol% H_2/Ar) ranging from 130 to 280 °C in 30 °C steps. The exposure time before the conditions were changed was ~30 min. The total flow was always 100 mL/min balanced in argon.

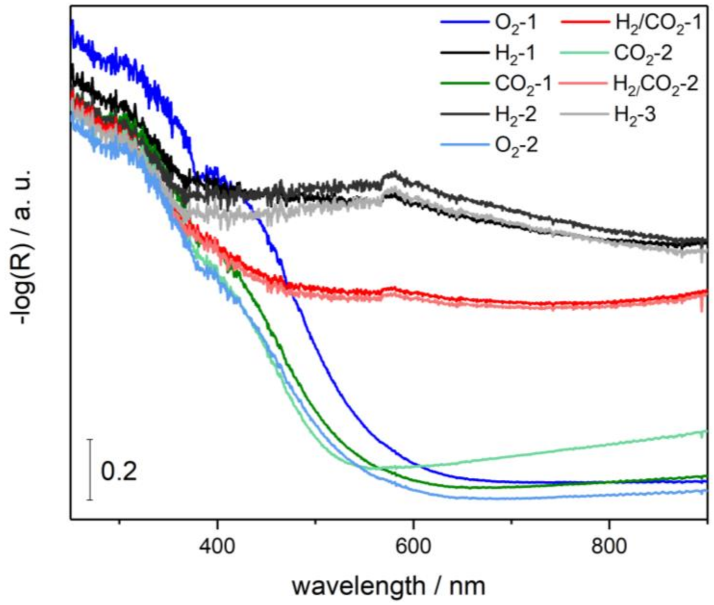

Figure S2. In situ/operando UV-vis spectra for c-In_2O_3 sheets recorded during the indicated gas exposures at 250 °C (total flow rate: 100 mL/min). Spectra were recorded for about 1 h after the gas exposure, except for the last O_2 phase, which was recorded after 30 min. The hyphenated numbers indicate how often the sample was exposed to the respective gas phase.

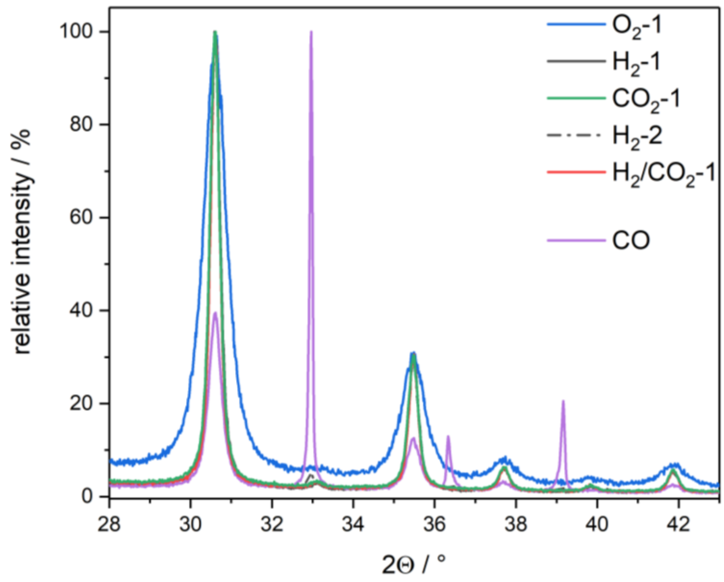

Figure S3. XRD results of c-In_2O_3 sheets recorded after the indicated gas exposures at 250 °C (total flow rate: 100 mL min^{-1}). The diffraction patterns were recorded sequentially in the following order: O_2, H_2, CO_2, H_2, H_2/CO_2. The diffraction pattern after the CO treatment was recorded after a prior O_2 treatment. The hyphenated numbers in the legend indicate how often the sample was exposed to the respective gas phase.

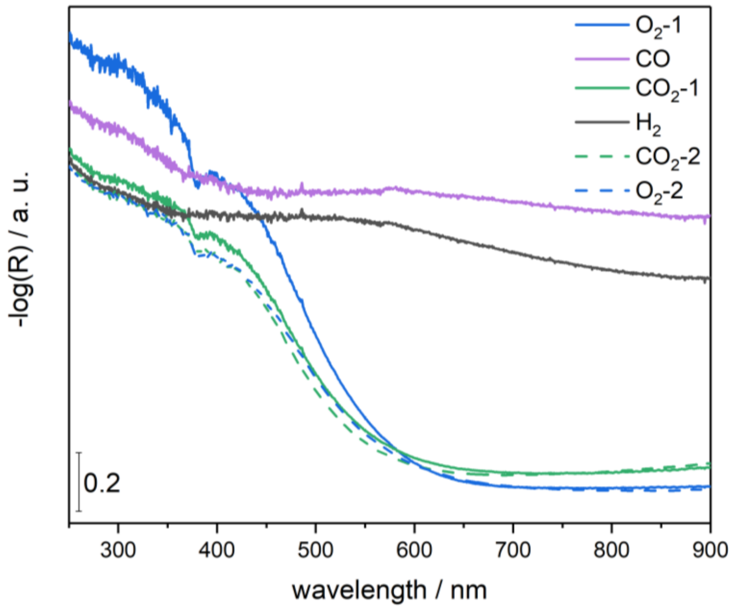

Figure S4. In situ UV-vis spectra for In_2O_3 sheets recorded during the indicated gas exposures at 250 °C and at a total flow rate of 100 mL min^{-1}. Spectra were recorded about for 1 h after the gas exposure. The hyphenated numbers in the legend indicate how often the sample was exposed to the respective gas phase.

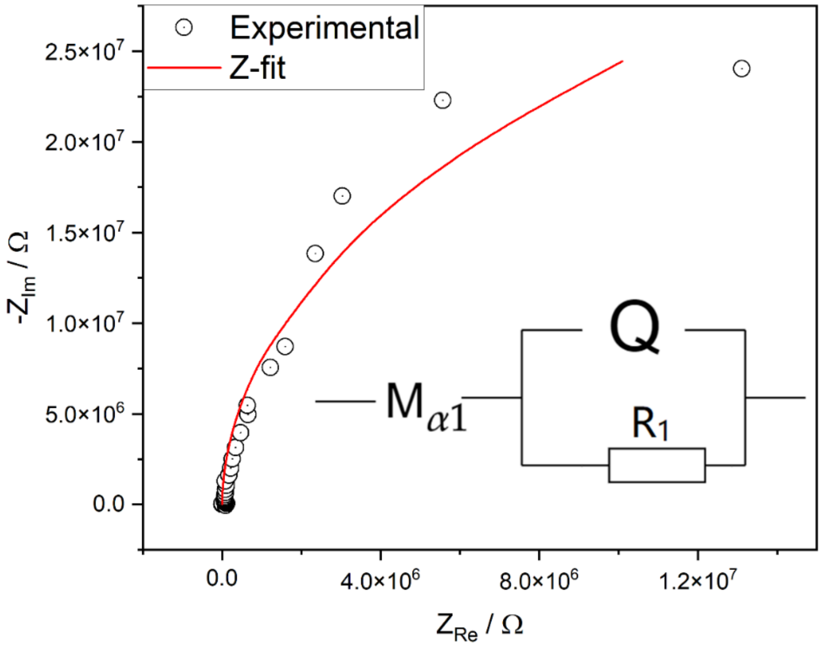

Figure S5. Nyquist plot based on operando p-EIS during the first O_2 (25 vol%; total flow 50 mL min^{-1}) treatment at 250 °C together with the results of a fit analysis using a modified restricted diffusion element $M_{\alpha 1}$. Q represents a constant phase element.

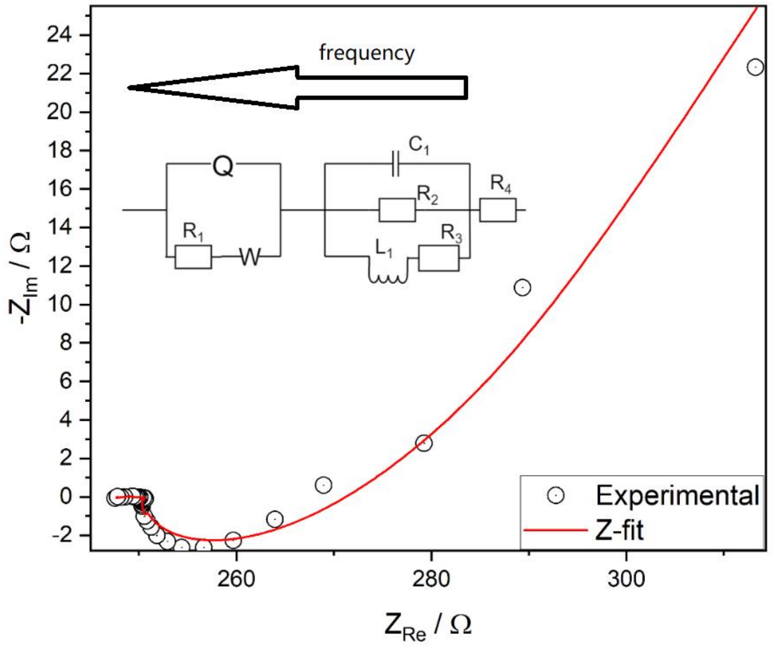

Figure S6. Nyquist plot based on operando p-EIS during the first H_2 treatment (4 vol%; total flow rate: 50 mL min^{-1}) at 250 °C with a pronounced contribution of the inductive impedance, arising from the percolation of gas into the sample's interior. Note that the p-EIS spectrum is inverted, meaning that the high frequencies are in the region of high resistance/reactance.

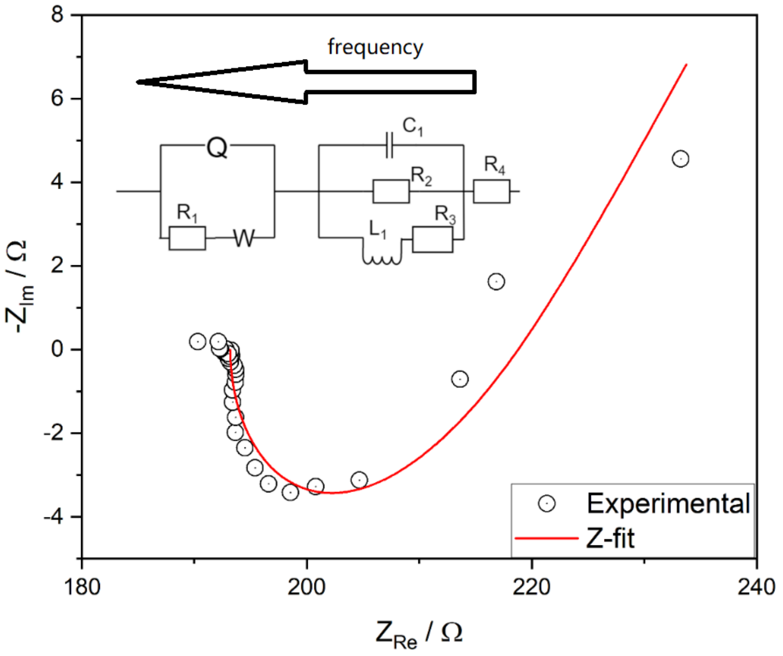

Figure S7. Nyquist plot based on operando p-EIS during the second H_2 treatment (4 vol%; total flow rate: 50 mL min^{-1}) at 250 °C with pronounced contribution of the inductive impedance, arising from the percolation of gas into the sample's interior. Note that the p-EIS spectrum is inverted here too, meaning that the high frequencies are in the region of high resistance/reactance.

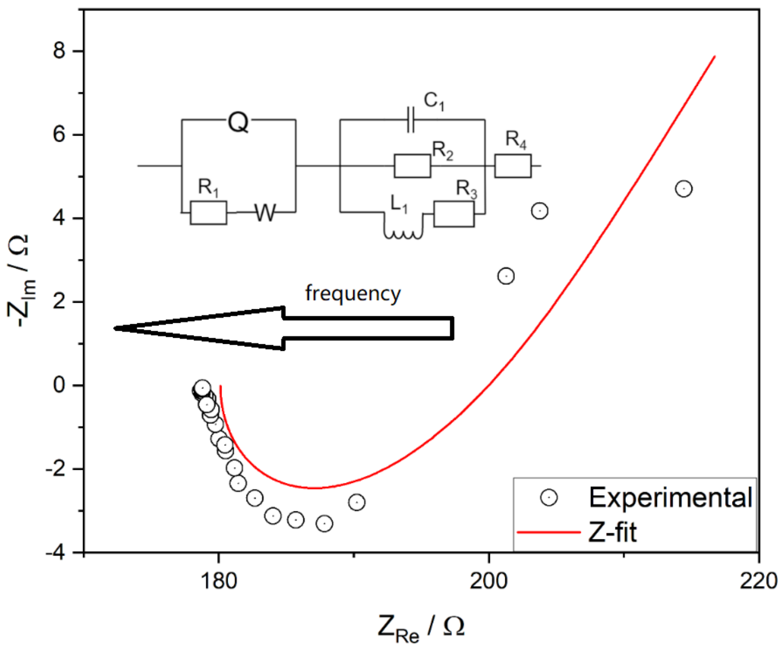

Figure S8. Nyquist plot based on operando p-EIS during the third H_2 treatment (4 vol%; total flow rate: 50 mL min^{-1}) at 250 °C with a pronounced contribution of the inductive impedance, arising from the percolation of gas into the sample's interior. Note that the p-EIS spectrum is inverted here, meaning that the high frequencies are in the region of high resistance/reactance.

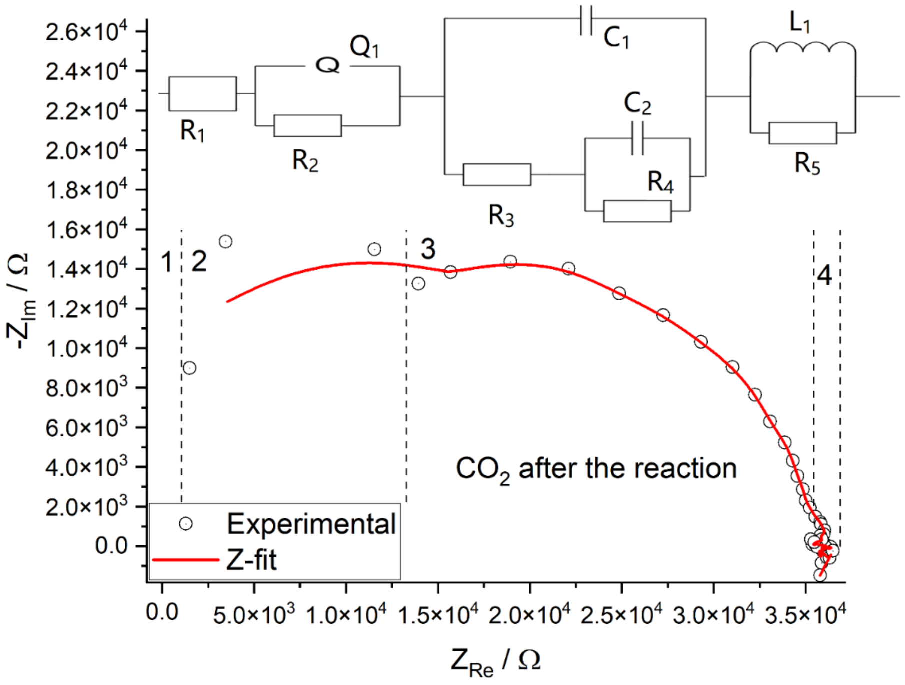

Figure S9. Nyquist plot based on operando p-EIS during the CO_2 treatment (2 vol%; total flow rate: 50 mL min^{-1}) after reaction conditions at 250 °C and the corresponding assignment in the equivalent circuit. Region 1: electrode bulk resistance; region 2: vacancy reorganization; region 3: capacitive behavior of the non-reduced In_2O_3; region 4: gas diffusion into In_2O_3.

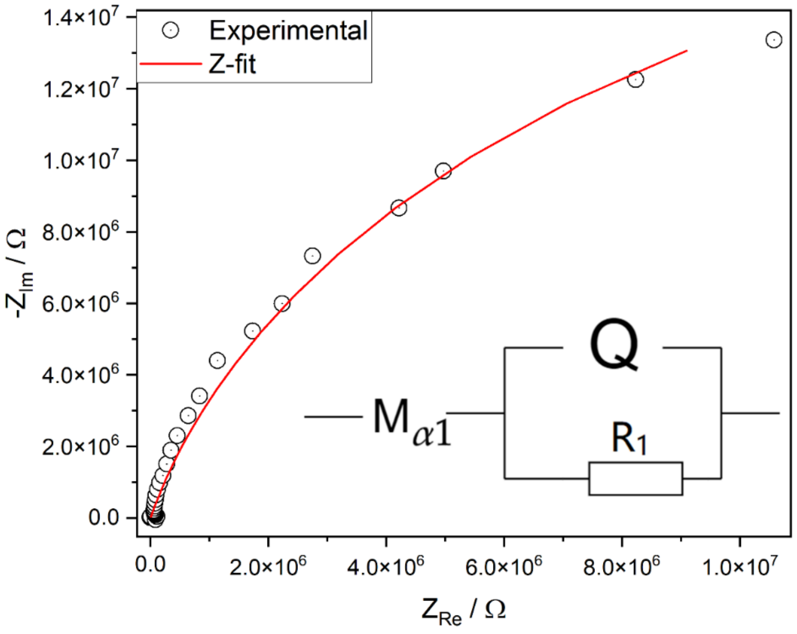

Figure S10. Nyquist plot based on operando p-EIS during the second O_2 treatment (25 vol%; total flow rate: 50 mL min^{-1}) at 250 °C together with the results of a fit analysis using a modified restricted diffusion element.

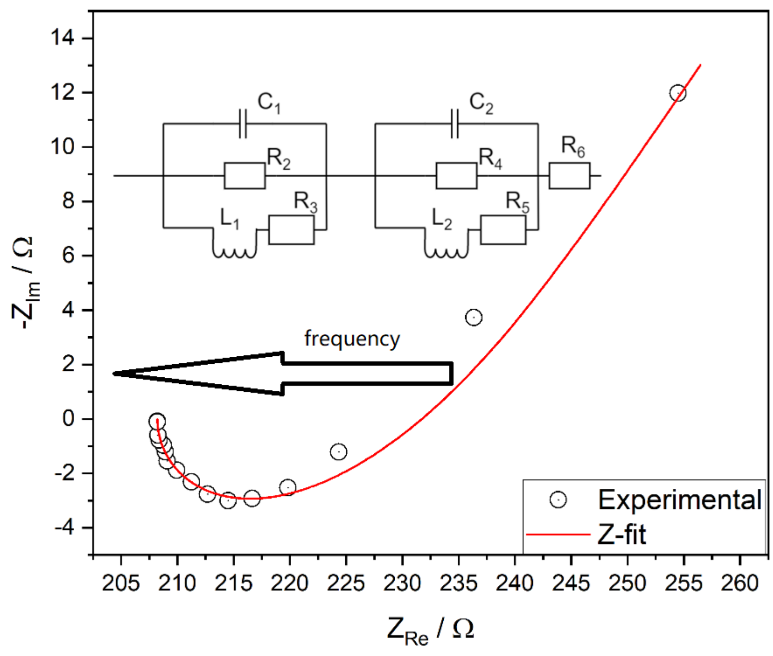

Figure S11. Nyquist plot based on operando p-EIS during the second exposure to reaction conditions (2 vol% CO_2, 4 vol% H_2; total flow rate: 50 mL min^{-1}) at 250 °C together with the results of a fit analysis using a modified restricted diffusion element. Note the absence of the pure-metallic phase (no horizontal line preceding the measurement point below 210 Ω), unlike the H_2-treatment in Figure S6. The experimental data shows a reversed behavior, where high-frequency points are located in the right part of the spectrum. R: resistance, C: capacitance, L: inductance. Q: non-ideal capacitance.

Table S2. Parameters used in equivalent circuit modelling of In_2O_3 in the different gas atmospheres. R: resistance, Q: constant phase element, C: capacitance, L: inductance, Rd_1: diffusion resistance, t: diffusion time, g: proportionality factor of the modified restricted diffusion element (O_2 treatments), s: inductive resistivity.

Equivalent circuit parameter/ Figure	CO_2 Fig. 3A	CO_2 after reaction Fig. S8	O_2^1 Fig. S4	O_2^2 Fig. S9	H_2^1 Fig. S5	H_2^2 Fig. S6	H_2^3 Fig. S7	Reaction 1 Fig. 3B	Reaction 2 Fig. S10
R_1	690.78 Ω	1501.68 Ω	-	-	-	-	-	13.41 Ω[a]	21.87 Ω[a]
Q_1	8.33E-8 F	1.31E-11 F	-	-	8.76F·s $^{(a-1)}$; a=1	2.08F·s $^{(a-1)}$; a=1	1.25F·s $^{(a-1)}$; a=1	-	-
R_2	1748 Ω	23527 Ω	-	-	3.66 Ω	5.62 Ω	5.76 Ω	3.31E-8 F	6.32E-7 F
C_1	1.5E-11 F	2.23E-11 F	-	-	2.98E-9 F	3.32E-8 F	3.58E-8 F	208.2 Ω	290 Ω
R_3	50427 Ω	3155 Ω	-	-	10.34 Ω	6.69 Ω	13.41 Ω	190 Ω	212 Ω
C_2	1.04E-7 F	2.93E-10 F	-	-	-	-	-	1.26E-8 F	0.78E-8 F
R_4	51490 Ω	3397 Ω	-	-	250.5 Ω	193.2 Ω	180.1 Ω	97.69 Ω	103.14 Ω
R_5	59191 Ω	3597 Ω	-	-	280.3 Ω[b]	137.8 Ω[b]	643.3 Ω[b]	41.7 Ω	52.9 Ω
L_1	4.47E-3 H	4.79E-3 H	-	-	-	-	-	1.61E-5 H	3.42E-5 H
Rd_1	-	-	13180 Ω	62590 Ω	-	-	-	-	-
t_1	-	-	2.54 s	0.907 s	-	-	-	-	-
g_1	-	-	0.183	0.687	-	-	-	-	-
g_2	-	-	1.32E-11	1.93E-11 F	-	-	-	-	-
R_2	-	-	68.29 Ω	43.94E-6 Ω	-	-	-	-	-
s_1	-	-	-	-	1.1E-9 $Ωs^{-1/2}$	-	-	-	-
L_2	-	-	-	-	2.03E-5 H	1.41E-6 H	1.29E-5 H	1.03E-5 H	2.2E-5 H

[a] Due to the fact that EIS spectra of the reaction are inverted and the equivalent circuit is therefore to be read from right to left, R_6 in Figure S10 corresponds to "R_1" here. [b] Similarly, in the case of H_2 exposure in Figs. S5-S7, R5 corresponds to R_1.

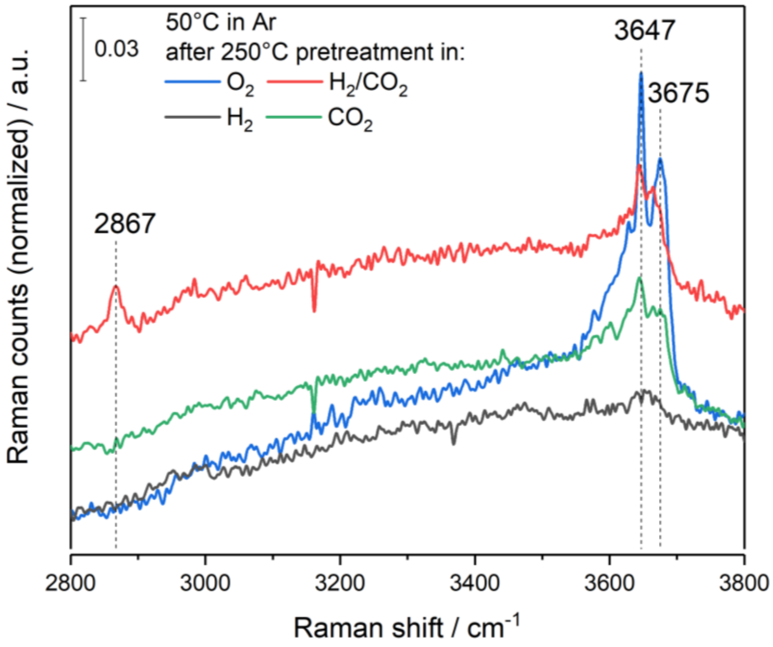

Figure S12. High wavenumber region of the quasi in situ Raman spectra, recorded in Ar at 50 °C after the indicated exposures at 250 °C (total flow rate: 100 mL min^{-1}). Spectra were normalized to the band at 306 cm^{-1} and recorded sequentially in the order O_2, H_2, H_2/CO_2, CO_2.

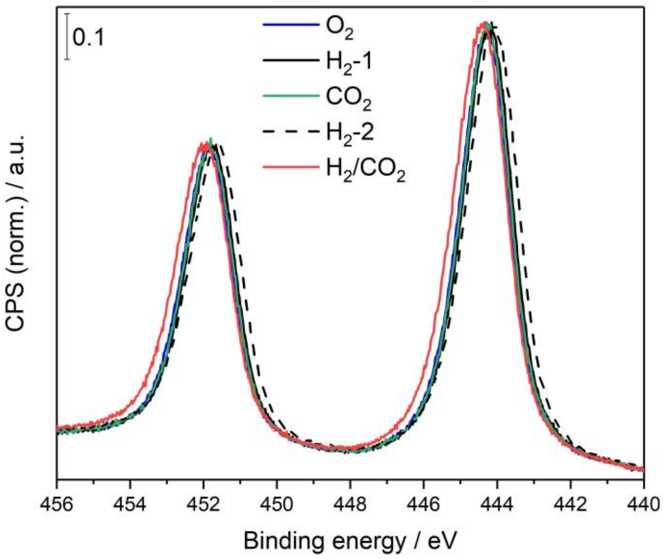

Figure S13. In 3d photoemissions spectra recorded quasi in situ after exposure to the indicated gas atmospheres at 250 °C and at a total flow rate of 100 mL min^{-1}. The blue spectrum was obtained after O_2 (25%) pretreatment, the gray ones after H_2 (4%) pretreatment, the green one after CO_2 (2%) pretreatment, and the red one after H_2/CO_2 (4%/2%) pretreatment. The gas sequence corresponds to that of the UV-vis experiments in Figure 2 up to the first reaction phase and the hyphenated number indicates the order of the gas sequence.

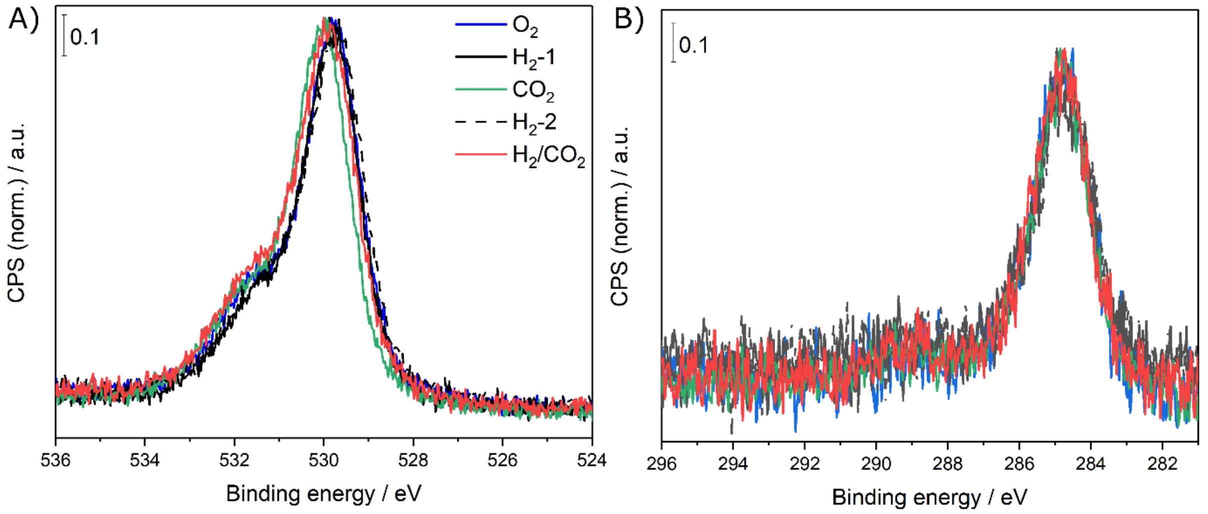

Figure S14. A) O1s and **B)** C1s photoemissions of In_2O_3 after exposure to the indicated gas atmospheres at 250 °C (total flow rate: 100 mL min^{-1}). The blue spectrum was obtained after O_2 (25%) pretreatment, the gray ones after H_2 (4%), the green one after CO_2 (2%), and the red one after H_2/CO_2 (4%/2%) pretreatment. The gas sequence corresponds to that of the UV-vis experiments in Figure 2 up to the first reaction phase and the hyphenated number in the legend indicates the order.

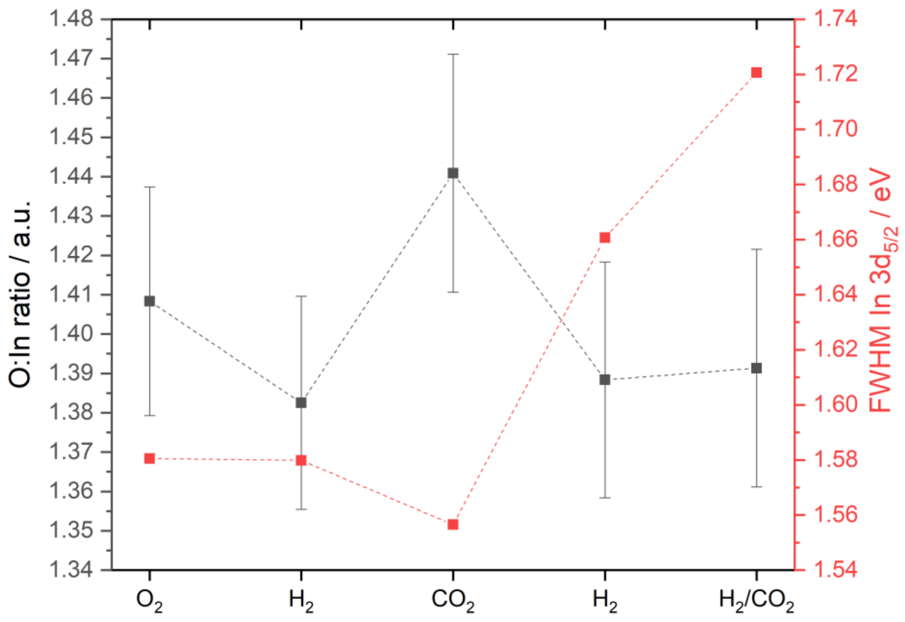

Figure S15. Linewidths (FWHM) of the In $3d_{5/2}$ signals from Figure 5 (red) and O:In ratios (black) determined from the quasi in situ XPS results. The dashed lines are guides to the eye.

Discussion of the quasi in situ XPS results

Figure S13 depicts the In 3d photoemissions, recorded after exposure to O_2, H_2, H_2/CO_2, and CO_2. After reaction conditions, a slight blue-shift in the binding energy (0.2 eV) and after the second H_2 pretreatment a slight red-shift are observed. The blue-shift may be indicative of the formation of surface hydroxides during reaction conditions and would be consistent with the findings from Raman spectroscopy, whereas the red-shift points to the formation of metallic indium species, indicating that metallic indium is also formed in small amounts at the surface besides its presence in the bulk, as shown by p-EIS and XRD analysis (see Fig. S2). These changes in the In 3d signals are also reflected in the corresponding linewidths (see Figure S15), revealing a significant increase in the FWHM after reaction conditions and the second H_2 exposure.

From the O:In ratios, shown in Figure S15, it can be seen that the surface is reduced under H_2 and oxidized under CO_2 (and O_2) atmosphere, in analogy to our other findings (see Figure 2). We attribute the observed behavior to variations in the number of oxygen vacancies and note that within the experimental uncertainty we do not detect differences between exposure to the CO_2 and O_2 phases. Finally, it should be mentioned that no significant changes can be seen in the O1s signals (see Figure S14A).

References

[1] M. Ziemba, L. Schumacher, C. Hess, *J. Phys. Chem. Lett.* **2021**, *12*, 3749–3754.

[2] C. T. Nottbohm, C. Hess, *Catal. Commun.* **2012**, *22*, 39–42.

[3] C. Schilling, C. Hess, *J. Phys. Chem. C* **2018**, *122*, 2909–2917.

[4] C. Schilling, C. Hess, *ACS Catal.* **2019**, *9*, 1159–1171.

[5] M. Ziemba, C. Hess, *Catal. Sci. Technol.* **2020**, *10*, 3720–3730.

[6] M. Ziemba, M. V. Ganduglia-Pirovano, C. Hess, *Faraday Discuss.* **2021**, *229*, 232–250.

[7] M. Ziemba, J. Weyel, C. Hess, *Appl. Catal. B* **2022**, *301*, 120825.

[8] C. Schilling, C. Hess, *Top. Catal.* **2017**, *60*, 131–140.

[9] C. Schilling, M. V. Ganduglia-Pirovano, C. Hess, *J. Phys. Chem. Lett.* **2018**, *9*, 6593–6598.

[10] D. Briggs, M. P. Seah, *Practical Surface Analysis by Auger and X-Ray Photoelectron Spectroscopy*, Wiley, Chichester, UK, **1983**.

[11] Z. Cai, Y. Ou, B. Zhang, J. Wang, L. Fu, M. Wan, G. Li, W. Wang, L. Wang, J. Jiang, et al., *J. Am. Chem. Soc.* **2021**, *143*, 3143–3152.
